# Postreproductive lifespans are rare in mammals

**DOI:** 10.1002/ece3.3856

**Published:** 2018-01-31

**Authors:** Samuel Ellis, Daniel W. Franks, Stuart Nattrass, Michael A. Cant, Destiny L. Bradley, Deborah Giles, Kenneth C. Balcomb, Darren P. Croft

**Affiliations:** ^1^ Centre for Research in Animal Behaviour University of Exeter Exeter UK; ^2^ Department of Biology University of York York UK; ^3^ Centre for Ecology and Conservation University of Exeter Penryn Campus Penryn, Cornwall UK; ^4^ Center for Whale Research Friday Harbor WA USA

**Keywords:** life history, menopause, postreproductive life, postreproductive stage, reproductive senescence, senescence

## Abstract

A species has a post‐reproductive stage if, like humans, a female entering the adult population can expect to live a substantial proportion of their life after their last reproductive event. However, it is conceptually and statistically challenging to distinguish these true post‐reproductive stages from the usual processes of senescence, which can result in females occasionally surviving past their last reproductive event. Hence, despite considerable interest, the taxonomic prevalence of post‐reproductive stages remains unclear and debated. In this study we use life tables constructed from published data on wild populations of mammals, and statistical measures of post‐reproductive lifespans, to distinguish true post‐reproductive stages from artefacts of senescence and demography in 52 species. We find post‐reproductive stages are rare in mammals and are limited to humans and a few species of toothed whales. By resolving this long‐standing debate, we hope to provide clarity for researchers in the field of evolutionary biology and a solid foundation for further studies investigating the evolution and adaptive significance of this unusual life history trait.

## 1 INTRODUCTION

An intuitive understanding of life history theory might lead to the prediction that the most effective way for an organism to maximize its fitness is to reproduce until the end of life. Contrary to this expectation, females of some species—notably humans—cease reproduction well before the end of life. The origin and evolution of female postreproductive lifespan have stimulated discussion and debate on the evolution of senescence, the selective forces impacting life histories, and the structure of human and nonhuman animal societies (Croft, Brent, Franks, & Cant, 2015; Hamilton, 1966; Hawkes & Coxworth, 2013; Johnstone & Cant, 2010; Williams, 1957). However, despite widespread interest, researchers are in disagreement about the taxonomic prevalence of extended postreproductive lifespans. Some studies suggest that postreproductive life is a common trait in mammals (Cohen, 2004; Finch & Holmes, 2010; Holmes & Ottinger, 2003; Nichols, Zecherle, & Arbuckle, 2016; Walker & Herndon, 2008), whereas others maintain that postreproductive lifespans are limited to humans and some species of toothed whale (Alberts et al., 2013; Austad, 1994, 1997; Foote, 2008; Levitis, Burger, & Lackey, 2013). This confusion has been caused by: (i) past difficulties in defining postreproductive lifespans (reviewed in (Levitis et al., 2013)) and (ii) using data from captive populations (discussed in (Croft et al., 2015)).

Defining postreproductive life is hindered by the conceptual difficulty of separating the postreproductive traits of interest from artifacts of senescence (Levitis et al., 2013). The postreproductive trait of interest is usually, either implicitly or explicitly, an extended postreproductive lifespan where females undergo menopause and terminate reproduction: called by Levitis et al. (2013) (and hereafter) a postreproductive stage. More formally, we define a species as having a postreproductive stage if a female entering the adult population can expect, on average, to live long enough to spend some of their life postreproductive. A great advantage of this definition is that this individual level trait can be scaled up to that of the population. In a population of females with postreproductive stages, a substantial proportion of females in the population will be postreproductive at any given time. This definition has clear ecological and evolutionary implications and can be unambiguously applied to taxonomically diverse species.

Aging theory predicts that in general the rates of senescence of physiological systems, including the reproductive system, are expected to be approximately simultaneous and proportional (Williams, 1957). In contrast, for a species to have a postreproductive stage, the processes of somatic and reproductive senescence need to have become decoupled to an extent that results in females regularly living beyond their reproductive lifespan for an extended period (Levitis et al., 2013). However—even in species without a postreproductive stage—natural variation in the relative timing of senescence of reproductive and somatic systems has the potential to result in some females occasionally living for a short time after their last reproductive event (termed postreproductive viability by (Levitis et al., 2013)). Senescence, along with chance and variation, can therefore result in some individual females in a population displaying short postreproductive lifespans. Such post reproductive viability has often mistakenly been referred to as akin to a true postreproductive stage in which the processes of somatic and reproductive senescence have become decoupled (e.g. Nichols et al., 2016). It is therefore important to distinguish the usual processes of senescence from true postreproductive stages.

Evidence of a postreproductive stage is often presented from captive populations. However, in many species, captive individuals have reduced increased survival because the risks of predation and starvation, and disease are greatly reduced (Tidière et al., 2016). Captivity can, therefore, extend rare and short postreproductive periods to mimic a postreproductive life history strategy (for examples of long postreproductive lifespans in captivity: (Cohen, 2004)). Captive breeding can also disrupt and shorten female reproductive lifespans compared to natural conditions (Hermes, Hildebrandt, & Göritz, 2004). However, these artificially prolonged postreproductive lifespans are the outcome of increased survival in captive conditions, not natural selection. Rather, the postreproductive lifespans observed in captive populations are an artifact of the low‐risk environment and the usual processes of senescence.

In this study, we compare patterns of reproductive and somatic senescence across fifty‐two wild mammalian populations and distinguish postreproductive life history strategies from the rare and short postreproductive lifespans that are artifacts of senescence. We do this using a population‐level measure: postreproductive representation (PrR) (Levitis & Lackey, 2011) which calculates the proportion of adult female years being lived by postreproductive females (Levitis & Lackey, 2011). Unlike other measures of postreproductive lifespan, PrR incorporates both the proportion of the population surviving to become postreproductive and their life expectancy upon becoming postreproductive (Levitis & Lackey, 2011), which provides a robust and statistically testable null hypothesis: that the proportion of adult female years being lived in the population is not statistically different than expected by chance. Moreover, PrR provides a measure that is directly comparable between species that differ in their total lifespans (Levitis & Lackey, 2011). Using PrR, we distinguish postreproductive life history strategies from artifacts of reproductive senescence and determine the prevalence of this unusual life history strategy in mammals. Using only data from wild animal populations, we avoid artifacts of artificially long lifespans that are observed in captive populations.

## 2 METHODS

### 2.1 Data

We constructed life tables for fifty‐two placental mammal species using published data on wild and unprovisioned populations (Table 1). We aimed to have as broad a taxonomic representation as possible among mammals, but age‐specific data are difficult to collect for wild animals. Hence, species with available data are usually long‐lived mammals of commercial, conservation, or scientific interest.

**Table 1:** Postreproductive representation (PrR) for 52 species of placental mammal (for simplicity defined and referred to as species rather than subspecies or ecotypes). PrR represents the proportion of adult female years being lived by postreproductive females. Asterix (*) shows those that are significantly different from 0 (*p* < .05). Ex at maturity is the expected lifespan for a female reaching sexual maturity. Age M is the age at which 95% of population lifetime fecundity has been reached, and Ex at maturity shows the expected lifespan of females who reach age M. Demography indicates the dispersal system for group living species, asocial represents species found in groups but without evidence of coherent social groups. Note: as postreproductive life expectancy scales with total lifespan, in short‐lived species there may be survival past the end of reproduction but on scales shorter than a year, so e_M_ will still be 0

Common Name	Species Name	Ex at maturity	Age M (95% Fecundity)	Ex at age M	PrR [Growing Population, Shrinking Population]	Demography	Refs
African elephant	*Loxodonta africana*	45	59	5	0.035	Male‐biased dispersal	(1, 2)
American bison	*Bison bison*	9	17	2	0.029 [0.009, 0.048]	Both sexes disperse	(3, 4)
American red squirrel	*Tamiasciurus hudsonicus*	3	8	0	0	Solitary	(5, 6)
Antarctic fur seal	*Arctocephalus gazella*	10	17	1	0.004 [0.001, 0.006]	Asocial	(7, 8)
Arctic fox	*Vulpes lagopus*	6	10	0	0.002 [0.001, 0.003]	Both sexes disperse	(9, 10)
Australian fur seal	*Arctocephalus pusillus*	11	20	0	0.002 [0.001, 0.003]	Asocial	(8, 11)
Banded mongoose	*Mungos mungo*	2	10	0	0	Limited dispersal by both sexes	(12, 13)
Belding's ground squirrel	*Urocitellus beldingi*	3	8	0	0.001	Male‐biased dispersal	(14, 15)
Bighorn sheep	*Ovis canadensis*	8	16	1	0.004	Male‐biased dispersal	(16, 17)
Blue monkey	*Cercopithecus mitis*	20	29	3	0.005	Male‐biased dispersal	(18, 19)
Brown bear	*Ursus arctos*	15	30	3	0.002 [0, 0.003]	Solitary	(20, 21)
Cheetah	*Acinonyx jubatus*	7	12	0	0.003	Solitary	(22, 23)
Chimpanzee	*Pan troglodytes*	29	50	4	0.006	Female‐biased dispersal	(18, 24)
Collared peccary	*Pecari tajacu*	9	15	0	0.005 [0.002, 0.008]	Male‐biased dispersal	(25, 26)
Eastern gorilla	*Gorilla beringei*	31	38	3	0.022	Mixed	(18, 27)
European badger	*Meles meles*	6	12	0	0.004	Mixed	(28, 29)
Fin whale	*Balaenoptera physalus*	22	95	13	0.006 [0, 0.012]	Solitary	(30, 31)
Golden‐mantled ground squirrel	*Callospermophilus lateralis*	2	7	0	0 [0, 0]	Solitary	(32, 33)
Hawaiian monk seal	*Monachus schauinslandi*	13	28	0	0	Asocial	(34, 35)
Himalayan tahr	*Hemitragus jemlahicus*	7	16	1	0.003 [0.001, 0.003]	Solitary	(36, 37)
Hippopotamus	*Hippopotamus amphibius*	31	41	2	0.009	Both sexes disperse	(38, 39)
Humans (Hadza hunter‐gathers)	*Homo sapiens*	59	41	26	0.443*	Female‐biased dispersal	(40–43)
Japanese macaque	*Macaca fuscata*	7	14	1	0.005	Male‐biased dispersal	(44, 45)
Japanese serow	*Capricornis crispus*	10	20	0	0 [0,0]	Both sexes disperse	(46, 47)
Killer whale	*Orcinus orca*	51	41	19	0.309*	Neither sex disperse	(48–50)
Lechwe	*Kobus leche*	6	11	0	0.003 [0.002, 0.006]	Both sexes disperse	(51, 52)
Leopard	*Panthera pardus*	9	16	1	0.012	Solitary	(53, 54)
Lion	*Panthera leo*	9	15	1	0.004	Male‐biased dispersal	(55, 56)
Long‐finned pilot whale	*Globicephala melas*	26	57	2	0.002 [0,0.002]	Neither sex disperse	(57, 58)
Meerkat	*Suricata suricatta*	3	12	0	0.004 [0.002, 0.008]	Male‐biased dispersal	(59, 60)
Moose	*Alces alces*	10	15	2	0.02 [0.007, 0.029]	Solitary	(61–63)
North American beaver	*Castor canadensis*	5	13	0	0.003 [0.002, 0.007]	Both sexes disperse	(64, 65)
Northern fur seal	*Callorhinus ursinus*	11	21	2	0.002 [0, 0.002]	Asocial	(66, 67)
Olive baboon	*Papio anubis*	13	23	2	0.02	Male‐biased dispersal	(45, 56)
Plains zebra	*Equus quagga*	12	19	1	0.006 [0.002, 0.011]	Both sexes disperse	(68, 69)
Polar bear	*Ursus maritimus*	13	27	3	0.013 [0.004, 0.019]	Solitary	(70, 71)
Pyrenean chamois	*Rupicapra pyrenaica*	6	11	0	0.001 [0.001, 0.001]	Male‐biased dispersal	(72, 73)
Raccoon	*Procyon lotor*	7	12	0	0.004 [0.002, 0.005]	Solitary	(74, 75)
Red deer	*Cervus elaphus*	12	17	0	0.001	Male‐biased dispersal	(76, 77)
Reindeer	*Rangifer tarandus*	8	16	0	0.001 [0, 0.002]	Both sexes disperse	(78–80)
Ring‐tailed lemur	*Lemur catta*	8	16	0	0.001	Male‐biased dispersal	(81, 82)
Short‐finned pilot whale	*Globicephala macrorhynchus*	38	34	13	0.26* [0.131*, 0.352*]	Neither sex disperse	(83, 84)
Soay sheep	*Ovis aries*	3	13	0	0.001	Male‐biased dispersal	(85, 86)
Steller sea lion	*Eumetopias jubatus*	14	27	2	0.017 [0.008, 0.029]	Asocial	(87, 88)
Verreaux's sifaka	*Propithecus verreauxi*	14	30	1	0.003	Male‐biased dispersal	(18, 82)
Walrus	*Odobenus rosmarus*	15	24	2	0.018 [0.008, 0.029]	Male‐biased dispersal	(89, 90)
Weddell seal	*Leptonychotes weddellii*	10	17	0	0.001 [0, 0.002]	Both sexes disperse	(91, 92)
West Indian manatee	*Trichechus manatus*	21	56	3	0.009 [0.003, 0.014]	Solitary	(93, 94)
White‐headed capuchin	*Cebus capucinus*	15	25	0	0.004	Male‐biased dispersal	(18, 95)
Yellow baboon	*Papio cynocephalus*	15	21	3	0.036	Male‐biased dispersal	(18, 45)
Yellow‐bellied marmot	*Marmota flaviventris*	5	12	2	0.006	Male‐biased dispersal	(96, 97)

Refs: 1. (Moss, 2001), 2. (Sukumar, 2003), 3. (Lott & Minta, 1983), 4. (Green, 1990), 5. (Larsen & Boutin, 1994), 6. (Descamps, Boutin, Berteaux, & Gaillard, 2008), 7. (Boyd, Croxall, Lunn, & Reid, 1995), 8. (Bonner, 1981), 9. (Angerbjörn, Hersteinsson, & Tannerfeldt, 2004), 10. (Eide, Stien, Prestrud, Yoccoz, & Fuglei, 2012), 11. (Gibbens, Parry, & Arnould, 2010), 12. (Cant, Nichols, Thompson, & Vitikainen, 2016), 13. (Mongoose Research Project, *pers comms*), 14. (Sherman, 1981), 15. (Sherman & Morton, 1984), 16. (Bérubé, Festa‐Bianchet, & Jorgenson, 1999), 17. (Festa‐Bianchet, 1991), 18. (Bronikowski et al., 2016), 19. (Cords, 1987), 20. (Schwartz et al., 2003), 21. (Bellemain, Swenson, & Taberlet, 2006), 22. (Kelly et al., 1998), 23. (Durant, Kelly, & Caro, 2004), 24. (Nishida & Hiraiwa‐Hasegawa, 1987), 25. (Low, 1962), 26. (Cooper et al., 2010), 27. (Stewart & Harcourt, 1987), 28. (Woodroffe, Macdonald, & da Silva, 1993), 29. (Carpenter et al., 2005), 30. (Mizroch, 1981), 31. (Aguilar, 2000), 32. (Bronson, 1979), 33. (Ferron, 1985), 34. (Job, Boness, & Francis, 1995), 35. (Harting, Baker, & Johanos, 2007), 36. (Caughley, 1966), 37. (Forsyth, Tustin, Gaillard, & Loison, 2004), 38. (Smuts & Whyte, 1981), 39. (Beckwitt et al., 2016), 40. (Marlow, 2004), 41. (Copeland et al., 2011), 42, (Lalueza‐Fox et al., 2011), 43. (Blurton Jones, 2016), 44. (Takahata et al., 1998), 45. (Melnick & Pearl, 1987), 46. (Akasaka & Maruyama, 1977), 47. (Miura, Kita, & Sugimura, 1987), 48. (Bigg et al., 1990), 49. (Olesiuk, Ellis, & Ford, 2005), 50. (Center for Whale Research *pers coms*.), 51. (Child & von Richter, 1968), 52. (Williamson, 1992), 53. (Balme et al., 2013), 54. (Fattebert, Balme, Dickerson, Slotow, & Hunter, 2015), 55. (Schaller, 1972), 56. (Packer, Tatar, & Collins, 1998), 57. (Martin & Rothery, 1993), 58. (Amos, Schlötterer, & Tautz, 1993), 59. (Sharp & Clutton‐Brock, 2010), 60. (Clutton‐Brock & Manser, 2016), 61. (Ericsson, Wallin, Ball, & Broberg, 2001), 62. (Solberg, Saether, Strand, & Loison, 1999), 63. (Gasaway, Dubois, Preston, & Reed, 1985), 64. (Payne, 1984), 65. (Busher, 2007), 66. (Lander, 1981), 67. (Insley, 2000), 68. (Grange et al., 2004), 69. (Fischhoff et al., 2007), 70. (Ramsay & Stirling, 1986), 71. (Ramsay, Stirling, Ramsey, & Stirling, 1988), 72. (Caughley, 1970), 73. (Loison, Jullien, & Menaut, 1999), 74. (Beasley & Rhodes, 2012), 75. (Hirsch, Prange, Hauver, & Gehrt, 2013), 76. (Benton, Grant, & Clutton‐Brock, 1995), 77. (Clutton‐Brock, Guinness, & Albon, 1982), 78. (Thomas & Barry, 1990a), 79. (Thomas & Barry, 1990b). 80. (Hirotani, 1990), 81. (Ichino et al., 2015), 82. (Kappler, 1999), 83. (Kasuya & Marsh, 1984), 84. (Heimlich‐Boran, 1993), 85. (Clutton‐Brock & Pemberton, 2004), 86. (Clutton‐Brock et al., 2004), 87. (Calkins & Pitcher, 1982), 88. (Loughlin, 2002), 89. (Born, 2001), 90. (Kastelein, 2002), 91. (Croxall & Hiby, 1983), 92. (Burns, Castellini, & Testa, 1999), 93. (Marmontel, 1995), 94. (Reynolds & Powell, 2002), 95. (Robinson & Janson, 1987), 96. (Schwartz, Armitage, & Van Vuren, 1998), 97. (Armitage, 1987).

We used both age‐specific survival and age‐specific fertility data to construct life tables. Data were collected from the literature searches in Google Scholar and Web of Science. As search terms, we used the species common and scientific names in conjunction with data‐specific terms such as “age‐specific fecundity/fertility,” “age‐specific mortality,” “reproduction,” “survival,” “age structure,” and “life table.” Data were used for analysis if the description of the population and methods were clear enough to be confident of their accuracy and interpretation. These types of age‐specific survival and fecundity data included in this analysis are described below.

### 2.2 Creating life tables: survival

Life tables are a widespread approach used to quantify life history in animals (e.g., Carey, 1993; Deevey, 1947; Erickson, Currie, Inouye, & Winn, 2006; Promislow & Harvey, 1990). At their simplest, life tables—in biology—are used to provide estimates of the rate of an animal's mortality and fecundity through their life. The construction of life tables therefore relies on deriving age‐specific estimates of survival and reproduction. The age‐specific data that we use to construct our life tables fall into three categories which we will call: longitudinal complete, longitudinal censored, and census data (Table 2). These three types of data are defined below.

**Table 2:** Summary of types of data used to construct the life tables used in this study. Superscript indicates the form of pregnancy data used to calculate *f*_x_, Y = observations of accompanying young, P = females were pregnant, P/B = combined pregnancy and birth data, and G = maternity of offspring inferred using genetic tools

	Longitudinal complete data	Single census data	Longitudinal censored data
Exact Ages	American red squirrel^Y^Bighorn Sheep^Y^Belding's ground squirrel^Y^Cheetah^Y^European badger^G^Hawaiian monk seal^Y^Leopard^Y^Lion^Y^Olive baboon^Y^Red deer^Y^Ring‐tailed lemur^Y^ Yellow‐bellied marmot^Y^	American bison^Y^Antarctic fur seal^P^Arctic fox^P^Australian fur seal^Y^Brown bear^Y^ Chamois^P/B^Collared peccary^P^Fin whale^P^Golden‐mantled ground squirrel^Y^Himalayan thar^P/B^Japanese serow^P/B^Lechwe^P/B^Long‐finned pilot whale^P^Meerkat^Y^Moose^Y^North American beaver^P^Northern fur seal^P^Polar bear^Y^Raccoon^P^Reindeer^P^Short‐finned pilot whale^P^Walrus^P^Weddell seal^Y^West Indian manatee^P^	Banded mongoose^P^Killer whale^Y^
Age Brackets		Hippopotamus^P/B^Plains zebra^Y^Steller sea lion^P^	
Survival/ Mortality	Japanese macaque^Y^Soay sheep^Y^		African elephant^Y^Blue monkey^Y^Chimpanzee^Y^Eastern gorilla Humans^Y^Northern muriqui^Y^Verreaux's sifaka^Y^White‐headed capuchin^Y^Yellow baboon^Y^

Longitudinal complete data require following all individuals for their entire lives. For wild populations, this is usually derived from long‐term field studies where animals born into the population are individually identifiable and tracked until death. In a longitudinal complete study, the exact year of birth and age at death are known. For each age category, the total number of individuals observed at age x (*N*_*x*_) is therefore known. From these data, other life table metrics can be derived (Carey, 1993; Krebs, 1998; Wachter, 2014) such as the probability of surviving to a given age (*l*_*x*_), the probability of surviving through an age (*p*_*x*_), and life expectancy at age x (*e*_*x*_). Fourteen of the species in the study have life tables calculated from longitudinal complete data (Table 2).

Longitudinal censored data area usually collected by long‐term studies, similarly to longitudinal complete data. However, unlike longitudinal complete, data ages of individuals are calculated or inferred for individuals born before the start of the study period, and individuals are not always followed until death (they are still alive at the end of the study period). Longitudinal censored data can therefore be both left and right censored which must be controlled for when calculating life table statistics (Carey, 1993; Wachter, 2014). Longitudinal censored data are most common for long‐lived species for which reliable age determination methods have been developed. Eleven species had life tables calculated based on longitudinal censored data (Table 2).

Census data are taken from a single survey (or multiple individual surveys) of the ages and reproductive state of individuals in a population. Surveys of populations can be based either on living or on dead individuals. The age and reproductive state of each individual in the survey are assessed. This can then be used to construct an age structure based on the number of individuals of each age found in the survey. Age structures from census data do not always monotonically decrease, due either to incomplete sampling or too short and/or long‐term deviations from a stable populations structures. Failure to account for this would lead to the biologically implausible conclusion that an individual's probability of surviving through a particular age is greater than one. To correct for this, we used variable bin widths (i.e., created an abridged life table (Wachter, 2014)): assigning individuals to age bins to create a monotonically decreasing age structure. These age bins were then used to estimate *N*_*x*_ (assuming mortality is equally spread through the binned range), which was in turn used to derive life tables (Krebs, 1998). This method assumes the population is at a stable age structure; an assumption violated if the population is growing or shrinking (Krebs, 1998). In the absence of detailed population growth data for most species, we model each species with census data under three growth scenarios: stable population (population growth (*r*) = 0), a population in serious decline (*r* = −0.1, approximately a decline of 10% per year), and a population in a period of rapid growth (*r* up to 0.1, the exact value depends on the species and some population growth scenarios are impossible for a given age structure). All life table statistics and derived statistics were calculated for all three population growth scenarios. Life tables for twenty‐seven mammal species in this study were based on census data (Table 2).

Age‐specific data were reported in the literature in three ways: as exact ages (38 of 52 species; Table 2), as binned age (three of 52 species; Table 2), and as derived survival or mortality data (11 of 52 species; Table 2). We converted binned ages to a predicted distribution of exact ages (*N*_*x*_) assuming mortality risk to be spread equally within each binned range. In some well‐studied species, derived life table values of survival (*l*_*x*_) or mortality (*q*_*x*_) were reported, and these values were used to directly calculate the full life table for those species.

Predation is a major source of mortality in animal populations, and in artificial predator‐free environments, individuals can have a higher survival than populations in entirely natural conditions. Three species in this study are from artificially predator‐free (but otherwise wild) populations—Himalayan tahr (*Hemitragus jemlahicus*), Pyrenean chamois (*Rupicapra pyrenaica*), and red deer (*Cervus elaphus*)—which may affect their demographic parameters and overestimate their PrR. Conversely, fin whales (*Balaenoptera physalus*) were hunted intensively during the period of modern whaling which increased mortality and is unlikely to have left the natural population parameters intact (Aguilar, 2000). The demographic parameters for fin whales should therefore be interpreted with caution.

### 2.3 Creating life tables: fecundity

In this study, we are interested in the presence or absence of female reproductive activity at a given ages rather than broader declines in fecundity with age. We therefore define fecundity as the proportion of reproductive females at a given age who are reproductively active. This definition is directly comparable between species because it does not depend on number of young produced per reproductive event, which can vary greatly between species. Reproductive and survival data were taken from the same population where possible, although data from the same population were published over multiple studies in some cases. Three main types of reproductive activity were used to estimate fecundity (*f*_*x*_): pregnancy, accompanying young, or genetic inference. Pregnancy is a direct measure of fecundity because pregnant females are, by definition, fertile and reproductively active (Table 2, superscript ^p^). Similarly, observations of a known age female accompanied by infants clearly demonstrate that the female is reproductively active (Table 2, superscript ^Y^). In some species, especially those based on a terminal sample, both pregnancy and young are combined into a single measure of fecundity (Table 2, superscript ^P/Y^). In a species breeding in shared burrows, parentage was inferred genetically after the emergence of the young (Table 2, superscript ^G^).

Because fecundity is reported as a proportion, it is vulnerable to small sample sizes returning highly variable changes in *f*_*x*_ values. This is a particular problem at later ages, when *N*_*x*_ is lower. To account for this, fecundity data were smoothed by weighting the magnitude in fecundity change between *x* and *x* + 1 by the number of individuals sampled at *x*+1.

### 2.4 Calculating postreproductive representation

PrR is calculated as the proportion of adult female years in the population being lived by postreproductive individuals (Levitis & Lackey, 2011). PrR is a population‐level measure and does not track the fecundity of individual females, rather it tracks fecundity of the population as a whole. The calculation of PrR incorporates both the probability of a female surviving to reproductive cessation and life expectancy once reproduction has ceased (equation 1). PrR is the ratio of female years lived by postreproductive females (*T*_*M*_) to the total years lived by adult females (*T*_*B*_). Throughout this article, following demographic convention, the subscript attached to a variable indicates the value of that variable at the subscripted integer age (Levitis & Lackey, 2011).

(1) $$\text{PrR}=T_{M}/T_{B}$$

Age M is the age at which 95% of population fecundity has been completed, independent of mortality (Levitis & Lackey, 2011). That is, age M represents the minimum age at which population fecundity (in our case total reproductive active females) of all females up to and including the age in question is greater than or equal to 95% of the total population fecundity of the total female population of all ages (equation 2). Ninety‐five percent of population is used to remove the influence of demographic outliers.

(2) $$\sum_{x=0}^{M}m_{x}\geq 0.95\sum_{x=0}^{\infty }m_{x}$$

Postreproductive years are calculated as the female years lived after age *M* (*T*_*M*_ = *e*_*M*_ * *l*_*M*_). Similarly, adult female years are usually defined as the female years lived after age B at which 5% of lifetime fecundity has been achieved (Levitis & Lackey, 2011). However, due to inconsistency in the reporting of early life survival in different species, we define age B as the youngest age at which females are observed reproducing in the species. Fixing age B allows consistent comparison between species. PrR is particularly suited for interspecific comparison because it is unitless and is therefore independent of the longevity of the species in question (Levitis & Lackey, 2011).

We also test the statistical significance of the calculated value of PrR for each species. As discussed above, the expectation of senescence is that the rates of aging of different biological systems are expected to be approximately simultaneous and proportional and shaped by the risks of extrinsic mortality (Williams, 1957). The null hypothesis is therefore that survival (*l*_*x*_)—the combined effect of intrinsic and extrinsic mortality on a population—and fecundity (*f*_*x*_) should decline at the same rate, that is, PrR = 0 (Levitis & Lackey, 2011). We test this by simulating 9999 populations of 1000 individuals in which this null hypothesis is true and comparing this to our observed data (Levitis & Lackey, 2011). Significance is calculated separately for each species by generating null populations based on that species’ demographic parameters. The reported *p* values (Table 1) indicate the number of times that this simulated PrR was greater than or equal to the observed PrR (with the sample included in the numerator and denominator; see equation 1 in (Ruxton & Neuhäuser, 2013)).

In natural conditions, the usual processes of senescence can result in rare and/or brief female survival past last reproduction. These populations will have a low PrR which is unlikely to be significantly different from that expected by chance. In contrast, for species with a postreproductive life history strategy, a large proportion of females will be postreproductive resulting in a high PrR, significantly different from zero (Levitis & Lackey, 2011; Levitis et al., 2013).

## 3 RESULTS

**Figure 1:**
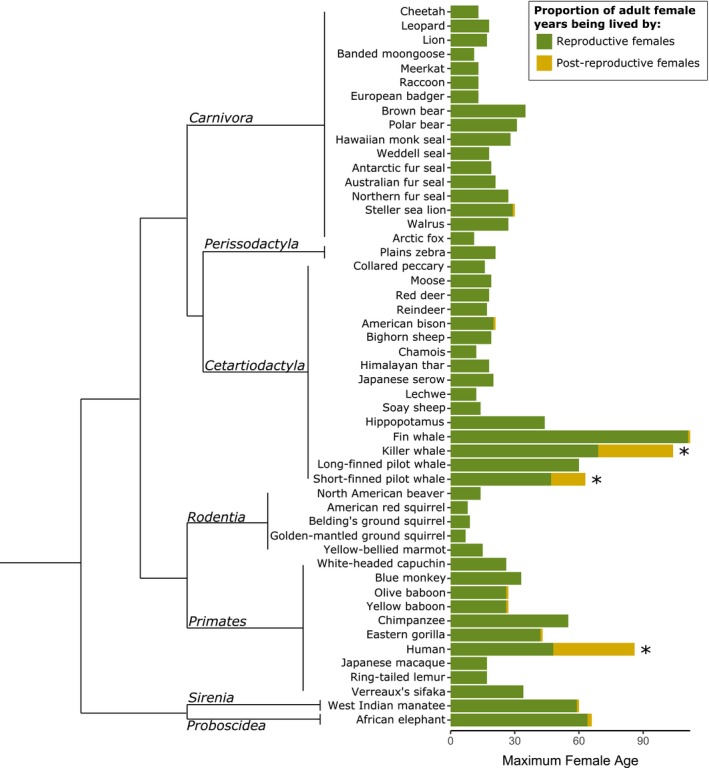
Proportion of female years in the population being lived by postreproductive individuals, scaled by maximum female age in 52 species of mammal. Each bar (right) shows the proportion of female years in the population being lived by reproductive (green) and post reproductive (orange) females. The length of the bar is equivalent to the maximum female lifespan of the species. A significant proportion of adult females years being lived by postreproductives is indicated by an asterisk (*). Species are ordered by family according to (Meredith et al., 2011) and within family alphabetically. Phylogeny (left) represents the relationships between mammalian orders (Meredith et al., 2011), branches are unscaled.

Three of the 52 mammal species have a postreproductive representation significantly greater than 0 (Figure 1; Table 1): humans (PrR = 0.43), killer whales (PrR = 0.34), and short‐finned pilot whales (PrR = 0.26 [0.13–0.35 (population decline‐population growth)]). For all the other 49 species of mammals, females did not have a postreproductive lifespan that differed from that expected by chance.

Females of all three species with evidence of a significant postreproductive stage have similar patterns of survival and reproduction. All three species have a comparable probability of living until the probable age of reproductive cessation (*lx* at *M*): humans = 0.59, killer whales = 0.73, and short‐finned pilot whales = 0.61. Similarly, in all three species, once a female has reached the probable age of last reproduction, they can expect to live a substantial number of years (*ex* at *M*): humans = 26 years, killer whales = 29 years, and short‐finned pilot whales = 13 years.

A striking feature of the measured mammalian postreproductive representation is their lack of variability. The PrR values are bimodal, species have either high postreproductive representation (greater than 0.25) or very low (not significantly different from 0). We find no intermediate values of PrR in the species examined; including in the species from artificially predator‐free populations (Himalayan tahr, Pyrenean chamois and red deer; Table 2).

## 4 DISCUSSION

There has been disagreement over the taxonomic prevalence of postreproductive stages with some authors suggesting that they are common (Cohen, 2004; Finch & Holmes, 2010; Holmes & Ottinger, 2003; Nichols et al., 2016; Walker & Herndon, 2008) and others suggesting that they are restricted to a small number of species (Alberts et al., 2013; Austad, 1994, 1997; Foote, 2008; Levitis et al., 2013). Our comparative analysis shows that postreproductive stages are rare in mammals and are confined to a limited number of species. In this study of 52 species of mammals, we report significant postreproductive stages in humans, killer whales, and short‐finned pilot whales. Some recent evidence also suggests that a third cetacean, false killer whales (*Pseudorca crassidens*), may also have a postreproductive stage (Photopoulou, Ferreira, Best, Kasuya, & Marsh, 2017). Far from being a common life history strategy, current evidence suggests that postreproductive stages are limited to humans and a few species of toothed whale.

Although our analysis shows that postreproductive life history strategies are rare in mammals, postreproductive viability may be more common. Postreproductive viability, survival after the end of reproduction, is indicated in many species by nonzero expected survival years after 95% of lifetime fecundity has been completed (e_B_ in Table 2). The apparent ubiquity of postreproductive viability underlines the importance of using appropriate methods to distinguish these short and rarely occurring artifacts of senescence from postreproductive life history strategies.

In this study, we have shown that in humans, killer whales, and short‐finned pilot whales, greater than 25% of adult female years in a population are being lived by postreproductive females. This is far beyond what is expected by the general process of senescence and is likely to be the result of active selection on female life history. Indeed in humans and killer whales—the two best‐studied species with a postreproductive stage—there is substantial evidence that the postreproductive stage has evolved in response to a trade‐off between both the inclusive fitness benefits and costs experienced by old females (Croft et al., 2015). In both humans and killer whales, older females provide benefits to the survival and reproduction of their offspring and grand‐offspring (Blurton Jones, 2016; Foster et al., 2012; Hawkes, O'Connell, Blurton Jones, Alvarez, & Charnov, 1998; Lahdenperä, Lummaa, Helle, Helle, & Russell, 2004). However, numerous examples of cooperative breeders demonstrate that the ability to help relatives does not alone lead to the evolution of postreproductive stages (Koenig & Dickinson, 2016). Humans and killer whales have social systems that might predispose females to evolve a postreproductive life history strategy. In ancestral humans, dispersal is thought to have been female‐biased (Copeland et al., 2011; Lalueza‐Fox et al., 2011; Marlow, 2004) and in resident ecotype killer whales, both males and females are philopatric remaining with their natal group for their entire life (Bigg, Olesiuk, Ellis, Ford, & Balcomb, 1990). Under both these dispersal systems, a females’ distant relatives are replaced with her offspring and grand‐offspring as she ages, increasing her average relatedness to her local group. These age‐related changes in local relatedness, kinship dynamics, can select for intergenerational conflict over reproduction (the reproductive conflict hypothesis (Cant & Johnstone, 2008)), which when taken together with the benefits of helping in late life, can select for the evolution of menopause (Cant & Johnstone, 2008; Johnstone & Cant, 2010). Under human and killer whale demography, reproductive conflict is predicted to select for harming behavior in early adulthood and helping behavior in late life (Cant & Johnstone, 2008; Johnstone & Cant, 2010). In killer whales, for example, older females lead their group at times of low resource abundance (Brent et al., 2015). Moreover, in both humans and killer whales, older females suffer costs by reproducing at the same time as their daughters, which will select for reproductive restraint and cessation in late life (Croft et al., 2017; Lahdenperä, Gillespie, Lummaa, & Russell, 2012).

Dispersal patterns, and their resultant kinship dynamics, are not enough in themselves to drive the evolution of a postreproductive stage. In this study, we see that mammals other than humans, killer whales, and short‐finned pilot whales have either female‐biased dispersal or bisexual philopatry but do not have a postreproductive stage (Table 1). The costs and benefits of helping relatives and ceasing reproduction are driven by older females being able to increase their inclusive fitness by aiding relatives (e.g., mother and grandmother effects (Hawkes et al., 1998)) and require a fitness cost of continued reproduction from intergenerational conflict (e.g., (Lahdenperä et al., 2012; Croft et al., 2017)). Without both these costs and benefits, postreproductive life histories are not expected to evolve, even given age‐related increases in local relatedness (Cant & Johnstone, 2008; Johnstone & Cant, 2010). The rarity of postreproductive life histories in mammals is likely to reflect the unusual behavioral and demographic circumstances required for it to be a beneficial strategy. It is also interesting to note that all three species we have found to have a postreproductive stage are relatively long‐lived (although importantly not all long‐lived species have postreproductive stages). More research is needed to establish if, for mammals, a relatively slow life history is a necessary condition for postreproductive stages to be beneficial.

Advances in our understanding of the evolution and processes of senescence (Lemaître & Gaillard, 2017; Nussey, Froy, Lemaitre, Gaillard, & Austad, 2013) have made it clear that rare and short survival beyond reproductive lifespan is not an adaptive strategy. Rather natural variation in the rate of senescence of various systems (reproductive and somatic) is likely to result in occasional and brief survival of females beyond their last reproductive event (Levitis et al., 2013). In contrast, the prolonged postreproductive life of female humans and some toothed whales is far beyond what we expect from the general processes of senescence (Levitis et al., 2013). Unlike previous studies investigating the taxonomic prevalence of postreproductive life histories, we have been able to differentiate both conceptually and statistically, postreproductive stages from senescence. In contrast to some previous studies (Cohen, 2004; Finch & Holmes, 2010; Holmes & Ottinger, 2003; Nichols et al., 2016; Walker & Herndon, 2008), we found postreproductive stages to be rare in mammals. This rarity is likely to reflect our conceptual and methodological separation of postreproductive stages from the natural process of senescence. In this study, we have clarified the taxonomic prevalence of postreproductive stages, allowing future studies to be put in an evolutionary context.

## COMPETING INTERESTS

We have no competing interests.

## AUTHOR CONTRIBUTIONS

SE and DPC conceived the project in discussion with DWF and MAC. SE, DLB, and DPC searched the literature to find the raw data for the analysis with assistance from MAC, DG, and KB. SE extracted and analyzed the data in discussion with DPC, DWF,and SN. SE wrote the first draft and constructed the figures with input from DPC; all authors provided input on subsequent drafts.

## DATA AVAILABILITY

This work is based on published material, and data are available in the paper cited in the text.
